# DMPS-induced neurological deterioration in neurological Wilson’s disease patients: a retrospective case-control study on clinical characteristics and risk factors

**DOI:** 10.3389/fneur.2025.1599209

**Published:** 2025-09-29

**Authors:** Yannan Gao, Jing Zhang, Lulu Tang, Shupei Jia, Guran Yu, Wenming Yang

**Affiliations:** ^1^Department of Neurology, The Affiliated Hospital of Nanjing University of Chinese Medicine, Nanjing, China; ^2^Department of Neurology, The First Affiliated Hospital of Anhui University of Chinese Medicine, Hefei, China

**Keywords:** Wilson’s disease, neurological deterioration, risk factors, clinical characteristics, sodium dimercaptopropanesulfonate

## Abstract

**Background and aim:**

Wilson’s disease (WD), an autosomal recessive copper metabolism defect, causes pathological copper deposition in hepatic and neurological systems, culminating in cirrhosis and neuropsychiatric manifestations. Our understanding of neurological deterioration in neurological WD patients following sodium dimercaptopropanesulfonate (DMPS) treatment is limited. Thus, this study aims to analyze the phenotypic spectrum and predictors of DMPS-induced neurological deterioration in neurological WD.

**Methods:**

Demographic (age, gender, weight), clinical (K-F ring, duration of illness), and biochemical parameters [alanine aminotransferase, aspartate aminotransferase, albumin, serum ceruloplasmin, blood urea nitrogen, serum creatinine, 24 h urinary copper, lactate, homocysteine (HCY)] were systematically evaluated alongside neuroimaging data, followed by receiver operating characteristic (ROC) curve analysis to identify predictive biomarkers for neurological deterioration in DMPS-induced neurological WD patients.

**Results:**

A total of 277 neurological WD patients were enrolled, among whom 24.5% (68/277) developed neurological deterioration. Notably, 70.6% (48/68) of the patients experiencing neurological worsening were male. Among the patients, 91.2% (62/68) exhibited mild deterioration, while 8.8% (6/68) experienced severe deterioration. Multivariate logistic regression analysis indicated that sex [odds ratio (OR) = 0.41[95% confidence interval (CI) = 0.18–0.94], *p* = 0.035], brain Magnetic Resonance Imaging (MRI) score (OR = 2.89[95% CI = 1.99–4.21], *p* < 0.001), and HCY (OR = 1.45[95% CI = 1.27–1.65], *p* < 0.001) were associated with neurological deterioration. Subgroup analysis revealed statistically significant differences in male proportion (36/19 vs. 75/84, *p* = 0.019), brain MRI score (median: 5 vs. 4, *p* < 0.001), and HCY levels (mean: 20.75 vs. 17.77, *p* < 0.001) between the deterioration and non-deterioration groups within the under-35 cohort. ROC analysis of composite biomarkers demonstrated significant predictive capacity for neurological deterioration in DMPS-induced neurological WD (AUC = 0.862).

**Conclusion:**

Neurological deterioration in DMPS-induced neurological WD patients is not rare and predominantly occurs in males. We identified three independent risk factors for this deterioration: sex, brain MRI score, and HCY. A composite risk model incorporating these parameters achieved superior predictive accuracy compared to individual biomarker.

## Introduction

Wilson’s disease (WD), an autosomal recessive disorder resulting from pathogenic *ATP7B* mutations, disrupts copper homeostasis with toxic hepatic, cerebral, and corneal accumulation. The prevalence of this disorder in the Chinese population is approximately 5.87 in 100,000, with a higher incidence observed in younger individuals (1). Clinically, the disease primarily manifests with symptoms of hepatic impairment and neurological deficits, the latter of which include dystonia, tremors, bradykinesia, ataxia, cognitive impairments, and chorea-like movements. The use of copper chelators to reduce copper overload has been shown to be an effective method for improving neurological symptoms in WD patients (2). However, approximately 50% of patients with neurological WD continue to experience neurological symptoms during the copper chelation process (3, 4), and more than 30% of these patients experience neurological deterioration during the early stages of chelation therapy (5, 6).

Sodium dimercaptopropanesulfonate (DMPS) is a first-line copper chelator widely used among Chinese patients, exhibiting a copper-chelating efficacy 2.6 times greater than that of D-penicillamine (7). DMPS therapy has demonstrated efficacy in ameliorating hepatic dysfunction and neuropsychiatric manifestations, leading to its incorporation into the Clinical Practice Guidelines for WD in China (2022 edition) as an evidence-based recommendation for therapeutic decision-making. However, a proportion of WD patients experience paradoxical neurological decline following DMPS treatment, which can even be life-threatening. Zhang et al. (6) previously reported that WD patients undergoing DMPS therapy exhibited neurological deterioration, including tremors and speech disorders. This adverse reaction significantly affects the applicability of the intervention in specific populations. Furthermore, there is limited research on neurological deterioration associated with DMPS therapy. Therefore, the identification of predictive risk factors is essential for formulating individualized therapeutic approaches and mitigating the risk of neurological deterioration in WD patients.

This study explores clinical characteristics and risk factors of DMPS-induced neurological deterioration in neurological WD patients, offering an objective reference for clinical strategies. Identifying these risk factors can guide clinical decisions and patient management, emphasizing the need for personalized approaches in complex conditions.

## Materials and methods

### Study population

This retrospective study analyzed 277 neurologically predominant WD patients undergoing DMPS therapy at the First Affiliated Hospital of Anhui University of Chinese Medicine (Sept 2021–Oct 2023). The inclusion criteria were as follows: (a) Satisfaction with the Leipzig scoring system diagnostic criteria for WD, the Leipzig score ≥4 (8); (b) Neurological symptoms as the initial presentation, neurological score ≥ 1 regarded as neurological WD (9, 10); (c) The clinical data were complete. The exclusion criteria were as follows: (a) Severe hepatic or renal dysfunction; (b) Switching of chelation agents during treatment; (c) Undergoing splenectomy or splenic artery embolization during treatment; (d) Severe psychiatric disorders encompassing severe depressive episodes (with psychomotor impairment), bipolar affective disorder (manic or mixed episodes), and profound cognitive impairment that significantly impacted daily functioning (11); (e)Forced cessation of chelation due to significant disease exacerbation during treatment; (f) Allergy to the trial medicine.

This study was designed as case–control study and was approved by the Ethics Committee of the First Affiliated Hospital of Anhui University of Chinese Medicine (Approval No.: 2021AH-60).

### Data collection

The clinical characteristics and laboratory biochemical indicators of patients were retrospectively collected using the Hospital Information System (HIS) system of the Information Center. These included age, weight, gender, duration of illness, Kayser-Fleischer (K-F) ring, serum ceruloplasmin (CER), alanine aminotransferase (ALT), aspartate aminotransferase (AST), blood urea nitrogen (BUN), serum creatinine (Scr), albumin (ALB), 24-h urinary copper, lactate, and homocysteine (HCY). Additionally, electronic medical records regarding clinical symptoms, signs, medication history, imaging data, and disease progression before and after treatment were also recorded.

In this study, we conducted a follow-up assessment of the recovery of patients with neurological deterioration. The follow-up began after the patients were discharged and lasted for 6 months, with assessments conducted every 3 months. Data were collected through telephone interviews, video calls via WeChat, and outpatient clinic visits, including patients’ survival status and disease recovery. Loss to follow-up was defined as the inability to contact the patient during the follow-up period. Definition of Recovery: “Recovery” was defined as a return to the patient’s pre-deterioration neurological baseline status, as assessed by the same neurologist using the Modified Young Scale (MYS) (12). Post-deterioration Management: Following neurological deterioration, the standard protocol involved immediate temporary suspension of DMPS, initiation of supportive therapy, and subsequent cautious reintroduction of chelation therapy at a reduced dosage once the patient had stabilized.

### Deterioration and non-deterioration identification

Clinical data were prospectively collected and independently assessed by two board-certified neurologists using standardized functional assessment scales at both admission and discharge time points. Based on changes in total scores, the patients were classified into deterioration group and non-deterioration group. The study employed the MYS to evaluate neurological function changes. The MYS has proven to be a valuable instrument in the clinical assessment of WD, effectively serving as a tool for monitoring disease progression and treatment response. This scale contains eight domains: language, oropharyngeal muscular dystonia, appendicular muscular dystonia, ataxia, tremors, chorea-like movements, gait status, and higher cortical functions. Each domain consists of two items, with a five-point scoring system for each item based on the severity of symptoms, ranging from 0 (mild) to 4 (severe). The total score was derived by summing the scores of all items. Treatment efficacy was assessed using changes in the total score of MYS compared to baseline, with an increase of more than 2 points indicating neurological deterioration (13). In the present study, an increase in total score of no more than 4 points was classified as mild deterioration, while an increase exceeding 4 points was classified as severe deterioration.

### Clinical management

Both groups of patients had completed diagnostic evaluation and subsequently received standardized therapeutic management for a period exceeding 2 weeks, with each treatment cycle consisting of continuous copper chelation therapy for 5 days followed by a 2-day drug cessation period to mitigate adverse reactions (7). During this period, zinc and calcium supplements were administered. All neurological WD patients strictly adhered to a low-copper diet. DMPS (No.: H31021514, each 2 mL contains 0.125 g, Shanghai Hefeng Pharmaceutical Co., Ltd., Shanghai) was initiated intravenously at a dose of 5–10 mg·kg^−1^·d^−1^, gradually increased to 15–20 mg·kg^−1^·d^−1^ within 1 week, with administration once daily (14), this dosage was maintained throughout the treatment period.

### Quantitative assessment of lesioned brain regions

Based on the quantification standards for Magnetic Resonance Imaging (MRI) abnormal signals established by Sinha et al., abnormal signals in WD were classified into high and low signals on T2-weighted images. The semi-quantitative evaluation of T2 sequence MRI findings can reflect and assess the pathological stages and severity of brain involvement in WD patients to a certain extent (15, 16). Using a 3.0 T MRI system, imaging specialists scored the regions of interest based on the extent of high signal (graded 0–3) and signal intensity (scaled 0–3) in WD patients. The sum of these two scores yielded brain MRI score, with higher scores indicating more severe damage (17).

### Statistical analysis

SPSS 26.0 (SPSSInc., Chicago, Illinois, United States) was used for data analysis. All results were presented as the mean ± standard deviation, as percentage, or as the median [P25, P75]. Differences between groups for continuous variables and categorical data were assessed using the t-test, Mann–Whitney U test, and Chi-square test, respectively. Univariate analyses were performed to identify candidate variables related to DMPS-induced neurological deterioration in WD patients (*p* < 0.05). Subsequently, multivariate logistic regression analysis was conducted to quantify the independent effects of significant predictors. Odds ratios (OR) and 95% confidence interval (CI) were calculated, and the results were considered statistically significant at *p* < 0.05. A forest plot was generated using GraphPad Prism 9.0 (GraphPad Software, Inc., San Diego, California, United States) to assess the differences among the parameters. Simultaneously, a subgroup analysis based on age characteristics was conducted for all WD patients to assess the credibility and accuracy of the study results. And we performed a 6-month follow-up on the deterioration group to assess the recovery rate after deterioration. Additionally, we employed multivariable logistic regression to develop a composite diagnostic model integrating key biomarkers, subsequently validating its discriminative capacity through receiver operating characteristic (ROC) curve analysis. The predictive accuracy was quantified by calculating the area under the curve (AUC) with corresponding 95% CI, alongside determination of optimal cutoff value maximizing the Youden index (J = sensitivity + specificity − 1). Diagnostic performance strata were defined as follows: AUC > 0.90 (excellent discrimination), 0.70–0.90 (moderate clinical utility), 0.50–0.70 (limited predictive value), and <0.50 (no better than chance prediction).

## Results

### Baseline data and clinical characteristics

As shown in Table 1, a total of 68 neurological WD patients with DMPS-induced neurological deterioration were included in the present study, accounting for 24.5% of the study population. Among the patients, 91.2% (62/68) exhibited mild deterioration, while 8.8% (6/68) experienced severe deterioration. Aged 10–52 years (mean, 26.38 ± 9.02 years), the 68 patients included 70.6% (48/68) males. Controls (n = 209) were WD patients without neurological deterioration, aged 11–52 years (mean, 28.51 ± 7.97 years), 45.9% (96/209) of whom were male. Both groups exhibited consistent biomarker profiles, characterized by positive K-F ring and serum CER levels below 200 mg/L. Moreover, the two groups demonstrated comparable characteristics with respect to age, ALT, AST, BUN, Scr, and ALB (*p* > 0.05).

**Table 1:** Baseline data and clinical characteristics between the two groups (x̅ ± s).

Variable	Deterioration group (*n* = 68)	Non-deterioration group (*n* = 209)	t/X^2^	*p* value
Age (year)	26.38 ± 9.02	28.51 ± 7.97	1.85	0.065
Sex (*n*, %)			12.50	**<0.001**
Male	48 (70.6%)	96 (45.9%)		
Female	20 (29.4%)	113 (54.1%)		
Weight (kg)	62.63 ± 11.27	59.79 ± 9.94	0.12	**0.049**
Duration of illness (year)	5.97 ± 4.32	4.26 ± 3.31	3.16	**0.002**
ALT (IU/L)	23.21 ± 9.88	25.52 ± 13.29	1.32	0.188
AST (IU/L)	24.24 ± 10.14	23.87 ± 10.13	0.26	0.796
BUN (mmol/L)	4.80 ± 1.43	4.91 ± 1.78	0.47	0.640
Scr (umol/L)	48.62 ± 8.41	50.50 ± 7.98	1.67	0.096
ALB (g/L)	39.95 ± 5.21	38.87 ± 5.79	1.37	0.172
Brain MRI score (point)	5.00 ± 0.93	3.94 ± 0.94	8.14	**<0.001**
24-h urinary copper (ug)	794.91 ± 304.68	720.46 ± 249.08	2.02	**0.044**
Lactate (mmol/L)	0.66 ± 0.13	0.61 ± 0.16	2.16	**0.032**
HCY (umol/L)	20.85 ± 3.56	17.73 ± 2.82	7.39	**<0.001**

ALT, alanine aminotransferase; AST, aspartate aminotransferase; BUN, blood urea nitrogen; Scr, serum creatinine; HCY, homocysteine. Bold values indicate statistical significance at *p* < 0.05.

However, the deterioration group exhibited a higher proportion of male (48/20 vs. 96/113, *p* < 0.001), as well as elevated levels in weight (mean: 62.63 vs. 59.79, *p* = 0.049), duration of illness (mean: 5.97 vs. 4.26, *p* = 0.002), brain MRI scores (mean: 5.00 vs. 3.94, *p* < 0.001), 24-h urinary copper (mean: 794.91 vs. 720.46, *p* = 0.044), lactate (mean: 0.66 vs. 0.61, *p* = 0.032), and HCY (mean: 20.85 vs. 17.73, *p* < 0.001). Additionally, the common neurological deterioration symptoms observed in patients with neurological WD included dysarthria (77.9%, 53/68), dysphagia (70.6%, 48/68), postural abnormalities (47.1%, 32/68), and limb tremors (44.1%, 30/68), choreic involuntary movements (30.9%, 21/68), ataxia (29.4%, 20/68), and psychiatric disorders (2.9%, 2/68).

In this study, we conducted follow-up assessments for WD patients with neurological deterioration over a period of 6 months. At 3 months, the recovery rate in the deterioration group was 76.5% (52/68). By 6 months, this rate increased to 89.7% (61/68). The four most frequent symptoms of improvement were as follows: tremor of the hands and feet, abnormal limb posture, gait abnormalities, and psychiatric disorders. Notably, there were no reported deaths by the end of the follow-up period.

### Multivariable analysis

**Figure 1:**
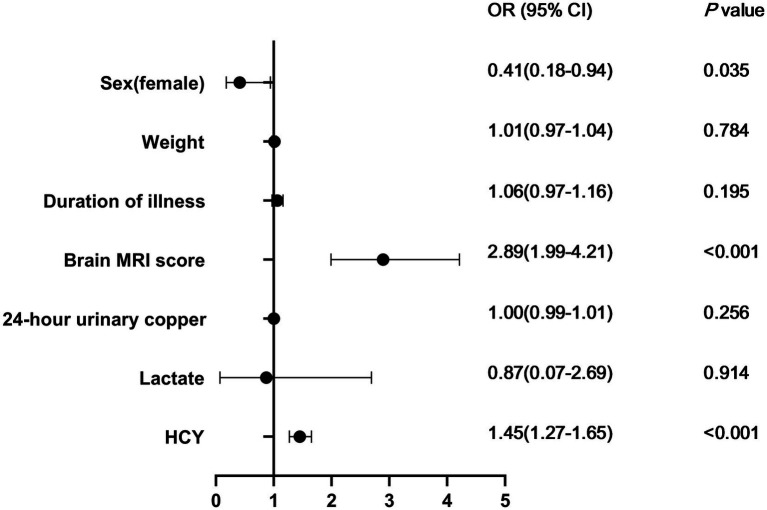
Presents a forest plot of each factor. The left column shows the factors. The odds ratio for each study is indicated by a solid circle, with the confidence intervals shown by horizontal lines. HCY, homocysteine.

Univariate analysis identified 7 parameters (including sex, weight, duration of illness, brain MRI score, 24-h urinary copper, lactate, and HCY) that were significantly associated with neurological deterioration (*p* < 0.05). These parameters were included in multivariate logistic regression model. Multivariate logistic regression analysis indicated that sex (OR, 0.41; 95% CI, 0.18–0.94; *p* = 0.035), brain MRI score (OR, 2.89; 95% CI, 1.99–4.21; *p* < 0.001), and HCY (OR, 1.45; 95% CI, 1.27–1.65; *p* < 0.001) were associated with neurological deterioration (Table 2; Figure 1).

**Table 2:** Multivariable logistic regression analysis of neurological deterioration risk factors (x̅ ± s).

Variable	Deterioration group (*n* = 68)	Non-deterioration group (*n* = 209)	OR (95% CI)	*P* value
Sex (*n*, %)			0.41 (0.18–0.94)	**0.035**
Male	48 (70.6%)	96 (45.9%)		
Female	20 (29.4%)	113 (54.1%)		
Weight (kg)	62.63 ± 11.27	59.79 ± 9.94	1.01 (0.97–1.04)	0.784
Duration of illness (year)	5.97 ± 4.32	4.26 ± 3.31	1.06 (0.97–1.16)	0.195
Brain MRI score (point)	5.00 ± 0.93	3.94 ± 0.94	2.89 (1.99–4.21)	**<0.001**
24-h urinary copper (ug)	794.91 ± 304.68	720.46 ± 249.08	1.00 (0.99–1.01)	0.256
Lactate (mmol/L)	0.66 ± 0.13	0.61 ± 0.16	0.87 (0.07–2.69)	0.914
HCY (umol/L)	20.85 ± 3.56	17.73 ± 2.82	1.45 (1.27–1.65)	**<0.001**

HCY, homocysteine; 95% CI, 95% confidence interval; OR, odds ratio. Bold values indicate statistical significance at *p* < 0.05.

### Subgroup analyses

Table 3 presented the baseline characteristics of WD patients in this subgroup. In the subgroup of patients aged <35 years, the deterioration group had a significantly higher male proportion, brain MRI score, and HCY level than the non-deterioration group (*p* < 0.05). For patients aged ≥35 years, brain MRI score and HCY level still showed significant differences between the two groups (*p* < 0.05).

**Table 3:** Characteristics associated with neurological deterioration in WD subgroups by age (x̅ ± s).

Variable	<35 years			≥35 years		
	Deterioration group (*n* = 55)	Non-deterioration group (*n* = 159)	*P* value	Deterioration group (*n* = 13)	Non-deterioration group (*n* = 50)	*P* value
Age (year)	23 (18, 30)	26 (21, 30)	0.059	38 (36, 41)	38 (36, 41)	0.693
Sex (*n*, %)			0.019			0.461
Male	36 (65.5%)	75 (47.2%)		4 (30.8%)	21 (42.0%)	
Female	19 (34.5%)	84 (52.8%)		9 (69.2%)	29 (58.0%)	
Weight (kg)	61.63 ± 11.60	59.67 ± 10.25	0.240	66.84 ± 8.92	62.16 ± 8.97	0.061
Duration of illness (year)	4 (1.5, 7)	3 (2, 6)	0.588	8 (6, 14)	4 (2, 6)	0.001
ALT (IU/L)	23.02 ± 10.09	24.87 ± 12.17	0.313	24.02 ± 9.25	27.60 ± 12.33	0.451
AST (IU/L)	22.94 ± 10.67	23.99 ± 9.81	0.505	29.73 ± 4.62	23.50 ± 11.17	0.055
BUN (mmol/L)	4.83 ± 1.41	4.74 ± 1.52	0.695	4.68 ± 1.59	5.47 ± 2.32	0.259
Scr (umol/L)	48.64 ± 8.09	50.74 ± 7.97	0.094	48.54 ± 9.99	49.74 ± 8.01	0.649
ALB (g/L)	40.29 ± 5.11	39.16 ± 5.77	0.199	38.51 ± 5.58	37.95 ± 5.78	0.758
Brain MRI score (point)	5 (4, 6)	4 (3, 5)	<0.001	5 (4, 6)	4 (3, 5)	0.004
24-h urinary copper (ug)	799.42 ± 318.81	730.71 ± 261.85	0.115	775.80 ± 245.85	687.85 ± 202.09	0.186
Lactate (mmol/L)	0.7 (0.6, 0.7)	0.6 (0.5, 0.7)	0.013	0.62 ± 0.16	0.60 ± 0.16	0.793
HCY (umol/L)	20.75 ± 3.77	17.77 ± 2.81	<0.001	21.27 ± 2.57	17.61 ± 2.89	<0.001

ALT, alanine aminotransferase; AST, aspartate aminotransferase; BUN, blood urea nitrogen; Scr, serum creatinine; HCY, homocysteine.

### Receiver operating characteristic curves for variables

**Figure 2:**
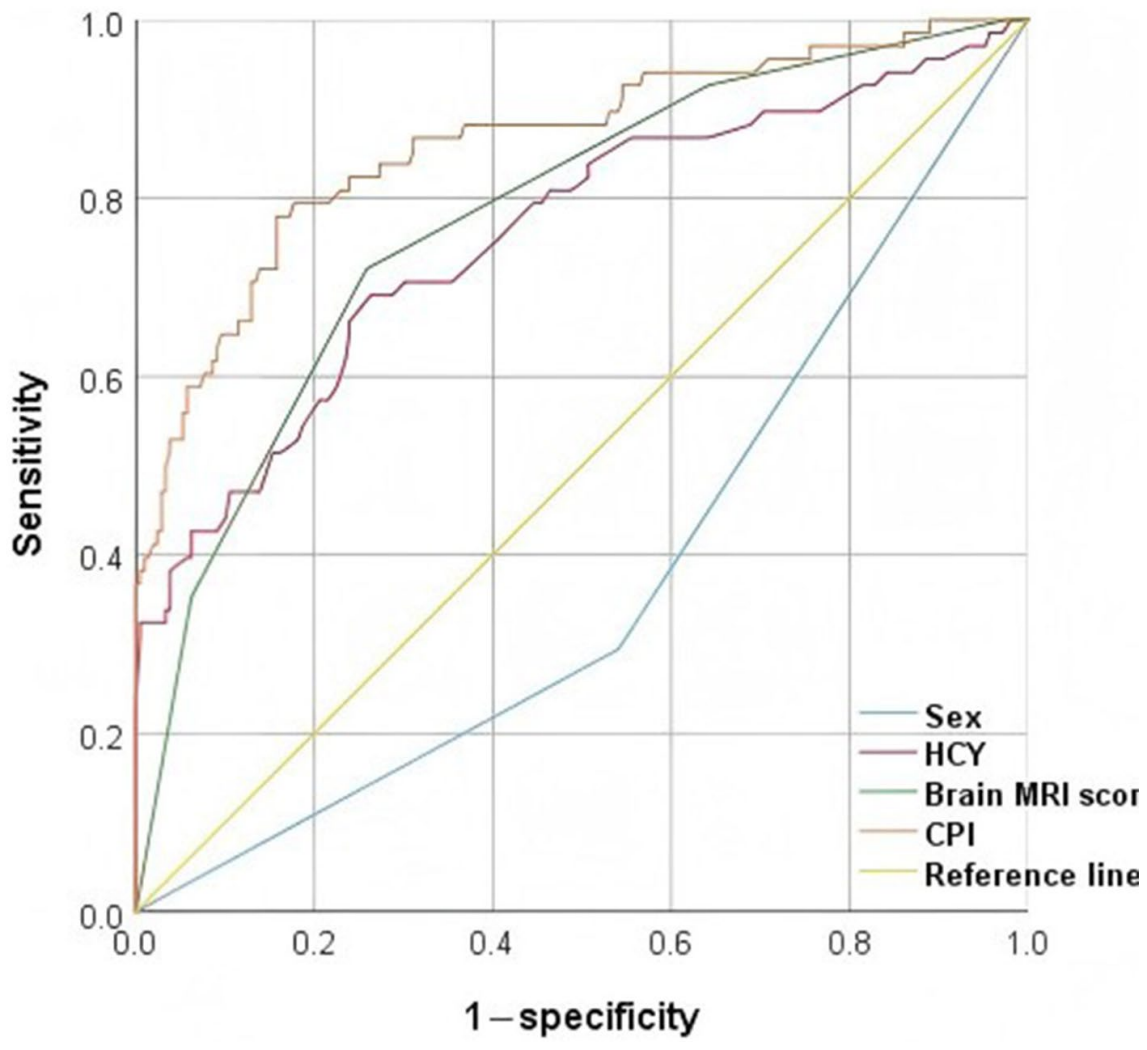
Presents an ROC curve evaluating the predictive performance of different variables for neurological deterioration, with true positive rate (sensitivity) plotted on the y-axis against false positive rate (1 − specificity) on the x-axis. The AUC quantifies the overall discriminative ability of each model. HCY, homocysteine; CPI, combined predictive indicators.

The ROC curves (Figure 2) for sex, brain MRI score, HCY, and combined predictive indicators (CPI) showed the AUC values of 0.377, 0.778, 0.758, and 0.862, respectively (Table 4). The result indicated that CPI had moderate clinical utility. In the ROC curve, the closer a point is to (0, 1), the better the model’s discriminatory ability. The predictive value of CPI for diagnosing DMPS-induced neurological deterioration in WD patients was superior to that of other individual indicators.

**Table 4:** Receiver operating characteristic curves for candidate variables.

Variable	AUC	Cut-off value	Sensitivity	Specificity	Asymptotic significance	95% CI	
						Lower limit	Upper limit
Sex	0.377	–	–	–	0.002	0.302	0.452
HCY	0.758	19.25	0.691	0.737	<0.001	0.686	0.830
Brain MRI score	0.778	4.5	0.721	0.742	<0.001	0.714	0.842
CPI	0.862	–	–	–	<0.001	0.806	0.917

HCY, homocysteine; CPI, combined predictive indicators.

## Discussion

In this retrospective study, we conducted a comprehensive analysis of 68 patients with WD who exhibited neurological deterioration following DMPS therapy, comparing their clinical profiles with 209 matched controls without neurological deterioration. Our findings demonstrated an overall incidence rate of 24.5% for DMPS-induced neurological deterioration, with mild neurological deterioration constituting the predominant presentation (91.2%), while severe deterioration was observed in only 8.8% of patients. Notably, the cohort with neurological deterioration showed statistically significant demographic distinctions: these patients presented a higher male predominance (male:female ratio 2.4:1 vs. 0.8:1 in controls) compared to the non-deterioration group. Our findings demonstrated a statistically significant concordance with the previous study by Cai et al. (18). It is speculated that the difference in estrogen levels may be the reason. Emerging evidence positions estrogen as a multifaceted neuroprotectant through three principal mechanisms: (a) potentiating endogenous antioxidant defenses via MAPK/ERK and PI3K/Akt signaling pathway activation; (b) suppressing neuroinflammatory responses through microglial deactivation mediated by TLR4/NF-κB signaling pathway; and (c) epigenetic modulation of neuroprotective gene networks involving pro-inflammatory cytokines, antioxidant enzymes, and anti-apoptotic proteins expression (19–21). These pleiotropic actions not only underlie its therapeutic potential in neurodegenerative disorders such as Alzheimer’s and Parkinson’s diseases but may also inform novel therapeutic strategies for mitigating neurological deterioration in WD, particularly given the observed gender disparity in disease progression trajectories.

Clinical analysis showed dystonia, movement disorders, and limb tremors were most frequent in WD patients with neurological deterioration, consistent with prior literature on neurological deterioration in WD patients treated with penicillamine (2, 22, 23). This indicates extrapyramidal symptoms are a main sign of neurological deterioration, closely linked to copper ions’ selective damage to the basal ganglia and its connecting fibers (24–26). Building upon the compelling evidence linking WD-associated neurological progression with copper dyshomeostasis, we hypothesize that neurological WD patients undergo pathogenic copper flux during clinical deterioration, which consequently triggers compartment-specific redistribution of copper ions through dysregulated transmembrane transport mechanisms. Therefore, we recommend a more cautious and individualized medication strategy and closer attention for neurological WD patients, which can improve their prognosis and quality of life.

Longitudinal follow-up data showed that in WD patients receiving DMPS therapy, the rate of neurological function recovery improved significantly in a time-dependent manner. By the 6-month follow-up point, the recovery rate reached 89.7%, with no treatment-related fatalities during the study period. Notably, compared to penicillamine, the continuous neurological deterioration rate was significantly lower with DMPS (27), indicating that DMPS-induced neurological deterioration may be partially reversible.

Multivariable logistic regression analysis revealed three independent predictors of neurological deterioration in neurological WD: sex (OR = 0.41, *p* = 0.035), brain MRI score (OR = 2.89, *p* < 0.001), and HCY (OR = 1.45, *p* < 0.001). While our analysis identified these biomarkers as independent risk factors, their association with underlying disease severity cannot be entirely ruled out. We further acknowledge that the initial neurological deterioration observed in some severe cases may represent a ‘therapeutic lag effect’, where neurological improvement is delayed despite successful biochemical decoppering. This finding is not consistent with the inclusion criteria applied in our study. Notably, in the subgroup of patients aged <35 years, the deterioration group had a significantly higher male proportion (36/19 vs. 75/84, *p* = 0.019), brain MRI score (median: 5 vs. 4, *p* < 0.001), and HCY level (mean: 20.75 vs. 17.77, *p* < 0.001) than the non-deterioration group. Meanwhile, for patients aged ≥35 years, brain MRI score (median: 5 vs. 4, *p* = 0.004) and HCY level (mean: 21.27 vs. 17.61, *p* < 0.001) still showed significant differences between the two groups. The absence of statistically significant sex-based differences in the ≥35-year age subgroup (*p* = 0.461) may be attributable to the limited sample size. While previous epidemiological studies have identified 5–35 years as the peak age range for WD onset (28, 29), critical gaps persist in type-specific epidemiological characterization. Motivated by this evidence gap, our study used 35 years as the age cutoff to analyze the clinical characteristics and risk factors of neurological deterioration in neurological WD patients.

The neurological deterioration in WD involves complex pathophysiological mechanisms, where the mislocalization, redistribution, and neurotoxicity of copper are closely linked (30–32). Copper accumulation in astrocytes compromises the blood–brain barrier, leading to neuronal and oligodendroglial damage and diverse neurological symptoms (33–35). Neuroimaging modalities, capable of directly quantifying end-organ neurological involvement through structural and functional correlates, serve as valuable objective biomarkers. This capability enables both guiding chelation therapy intensity based on real-time pathological burden assessment and predicting therapeutic responsiveness through baseline imaging signatures (25). T2-weighted imaging shows characteristic hyperintensity in the basal ganglia, thalamus, and/or brainstem in WD, seen in 90% of WD patients with neurological manifestations (36, 37). Building on this foundation, Dusek et al. demonstrated that WD primarily causes central atrophy through deformation and surface-based morphometry, with overall brain atrophy correlating with neurological severity (17). Although our study differs in perspective, it corroborates that quantitative MRI analysis offers further insights into WD-related brain damage and susceptibility changes.

HCY, a key mediator of oxidative stress, DNA epigenetic regulation, and cellular homeostasis, has been established as a biomarker for vascular and neurodegenerative disorders (38–40). While direct evidence linking HCY to neurological WD remains scarce, clinical studies have demonstrated significantly elevated serum HCY levels (*p* < 0.01) in WD patients with hepatic steatosis compared to those without fatty liver involvement (41). Mechanistic study using the Jackson toxic milk mouse model of WD reveals that copper accumulation disrupts hepatic methionine metabolism through inhibition of S-adenosylhomocysteine hydrolase, leading to pathological S-adenosylhomocysteine accumulation and inducing systemic hypomethylation, which triggers inflammatory cascades that ultimately contribute to neural tissue damage (42). These findings collectively suggest that HCY elevation may serve as a predictive indicator of neurological deterioration in WD, potentially reflecting copper-induced neuroinflammatory activation. Further investigations are required to delineate the molecular mechanisms linking HCY dysregulation to copper metabolism perturbations, with emphasis on their pathogenic crosstalk in WD progression.

Additionally, ROC curve analysis revealed that a multivariable-adjusted biomarker panel incorporating sex, brain MRI score, and HCY, demonstrated superior predictive performance for neurological deterioration in neurological WD (AUC = 0.862, *p* < 0.001). Therefore, timely assessment of these indicators is crucial for early identification of disease deterioration and improved prognosis. This not only deepens our understanding of the clinical features of neurological WD but also provides a more refined monitoring tool for clinical practice, thereby enhancing the precision of clinical interventions.

### Limitations

This study has several limitations. First, the retrospective design may introduce bias, and the single-center nature of the research could limit the generalizability of the findings to a broader population. Furthermore, while the sample size is adequate for preliminary analysis, it may not fully capture the spectrum of neurological manifestations in WD patients receiving DMPS treatment across different environmental factors influencing disease expression. The 6-month follow-up period, while aligned with standard early assessment windows for WD, may limit detection of delayed neurological events such as late-onset paradoxical deterioration or therapeutic lag effect. Future research should aim for multi-center, prospective studies with larger cohorts and extended follow-up durations to validate these findings. Additionally, exploring the potential mechanisms of neurological deterioration during chelation therapy and the long-term impact of DMPS treatment on neurological outcomes in WD patients is essential.

## Conclusion

Neurological deterioration in DMPS-induced neurological WD patients is not rare and predominantly occurs in males. We identified three independent risk factors for this deterioration: sex, brain MRI score, and HCY. A composite risk model incorporating these parameters achieved superior predictive accuracy compared to individual biomarker. Additionally, our findings not only provide clinicians with crucial data on neurological deterioration in WD but also revealed specific biomarkers for disease severity assessment, thus bolstering clinical decision-making.

## Data Availability

The original contributions presented in the study are included in the article/supplementary material, further inquiries can be directed to the corresponding authors.
